# Fluorescent cellulose aerogels containing covalently immobilized (ZnS)_x_(CuInS_2_)_1−x_/ZnS (core/shell) quantum dots

**DOI:** 10.1007/s10570-013-0035-z

**Published:** 2013-09-03

**Authors:** Huiqing Wang, Ziqiang Shao, Markus Bacher, Falk Liebner, Thomas Rosenau

**Affiliations:** 1 Key Laboratory of Natural Polymeric Materials and Application Technology, Department of Materials Science and Engineering, Beijing Institute of Technology, Zhongguancun South Street 5, Beijing, 10081 People’s Republic of China; 2 Division of Chemistry of Renewables, Department of Chemistry, University of Natural Resources and Life Sciences, Konrad-Lorenz-Straße 24, 3430 Tulln, Austria

**Keywords:** Cellulose, Aerogels, Photoluminiscence, Quantum dots, Ionic liquids

## Abstract

Photoluminiscent (PL) cellulose aerogels of variable shape containing homogeneously dispersed and surface-immobilized alloyed (ZnS)_x_(CuInS_2_)_1−x_/ZnS (core/shell) quantum dots (QD) have been obtained by (1) dissolution of hardwood prehydrolysis kraft pulp in the ionic liquid 1-hexyl-3-methyl-1*H*-imidazolium chloride, (2) addition of a homogenous dispersion of quantum dots in the same solvent, (3) molding, (4) coagulation of cellulose using ethanol as antisolvent, and (5) scCO_2_ drying of the resulting composite aerogels. Both compatibilization with the cellulose solvent and covalent attachment of the quantum dots onto the cellulose surface was achieved through replacement of 1-mercaptododecyl ligands typically used in synthesis of (ZnS)_x_(CuInS_2_)_1−x_/ZnS (core–shell) QDs by 1-mercapto-3-(trimethoxysilyl)-propyl ligands. The obtained cellulose—quantum dot hybrid aerogels have apparent densities of 37.9–57.2 mg cm^−3^. Their BET surface areas range from 296 to 686 m^2^ g^−1^ comparable with non-luminiscent cellulose aerogels obtained via the NMMO, TBAF/DMSO or Ca(SCN)_2_ route. Depending mainly on the ratio of QD core constituents and to a minor extent on the cellulose/QD ratio, the emission wavelength of the novel aerogels can be controlled within a wide range of the visible light spectrum. Whereas higher QD contents lead to bathochromic PL shifts, hypsochromism is observed when increasing the amount of cellulose at constant QD content. Reinforcement of the cellulose aerogels and hence significantly reduced shrinkage during scCO_2_ drying is a beneficial side effect when using α-mercapto-ω-(trialkoxysilyl) alkyl ligands for QD capping and covalent QD immobilization onto the cellulose surface.

## Introduction

Aerogels are fascinating functional materials that consist of coherent open-porous networks of loosely packed, bonded particles or fibers and additionally feature very low densities at high specific surface area (Liebner et al. 2013). They can be obtained from quite a broad range of precursors provided that the latter have the ability to form colloid dispersions from solution state (sol) with particles aggregating to macroscopic networks (gel), the voids of which are filled with the respective solvent (hydro- or solvogel). Subsequent replacement of the interstitial liquid by air converts the latter into aerogels which is typically accomplished by freeze-drying or drying with CO_2_ under supercritical conditions (scCO_2_).

Biopolymers, such as cellulose, starch, pectin, alginate, chitin, chitosan, carrageenan, and agar, have recently moved into the limelight of aerogel research (Jin et al. 2004; Pinnow et al. 2008; Hoepfner et al. 2008; Gavillon and Budtova 2008; Cai et al. 2008; Barud et al. 2008; Liebner et al. 2011, 2010; Sescousse et al. 2011). This is partly due to the increasing public awareness for renewable sources in general, but mainly the recognition of their unique chemical architecture that gives access to high-performance functional materials with unique properties has been the driving force.

Within a short decade of systematic research on cellulosic aerogels only, this new sub-class of aerogels has enormously advanced. Different technological approaches for the preparation of highly porous gel network structures of controlled morphology and their subsequent conversion into aerogels under full preservation of porosity have become available, and utilization of different cellulose sources (microcrystalline-, nanofibrillated-, bacterial cellulose, different types of pulp etc.), reinforcement strategies (cross-linking, interpenetrating networks, etc.), surface modification and functionalization have been introduced as important tools to control the properties of aerogels. Intriguing features such as densities ≤8 mg cm^−3^ (Liebner et al. 2010), low heat transmission, high interconnected porosity (≤99.99 %) and void surface area (≤650 m^−2^ g^−1^) render cellulose aerogels promising materials for a large variety of technical applications. Potential fields of use are high-performance thermal insulation (Plawsky et al. 2010), lightweight construction materials (Granstrom et al. 2011), oil–water separation (Cervin et al. 2012), photo-switchable (Kettunen et al. 2011) or shape-recovering superadsorbers (Zhang et al. 2012), bio-inspired cargo carriers on water and oil (Jin et al. 2011), adsorption of pollutants from air and water, catalysis (Koga et al. 2012), energy storage (Razaq et al. 2012; Hu et al. 2013), temporary templates (Korhonen et al. 2011), hemodialysis (Carlsson et al. 2012), controlled drug release in wound treatment (Haimer et al. 2010), or regenerative therapies where cellulosic aerogels have been studied as artificial blood vessels (Klemm et al. 2001), cartilage tissue (Bodin et al. 2007), or cell scaffolding materials (Liebner et al. 2011). Covalent immobilisation of quantum dots on the large inner surface area of cellulosic aerogels is a new approach that is considered to further expand the application potential of cellulosic aerogels.

Quantum dots (QD) are colloidal, mostly semiconductor-based nanoparticles of a size being typically equal to or smaller than the exciton Bohr radius (ca. 2–15 nm). At such small dimensions continuous band structures, such as those in bulk semiconductors, are no longer possible as quantum confinement of excited electron–hole pairs (“excitons”) causes quantization of energy levels (Zhang and Clapp 2011).

The multifaceted response of QDs towards photons of different energy (low energy such as UV, visible light, NIR radiation: photoabsorption or photosensitation; high energy such as X-rays or γ-rays: photoelectric ionization or photon annihilation on an atom nucleus and generation of an electron–positron pair) render QDs very interesting materials for a wide range of applications (Juzenas et al. 2008). In particular their optical properties that can be easily tuned by either variation of particle size and composition and/or subsequent surface modification are currently comprehensively studied. Different from conventional fluorophores, QDs have broad absorption spectra allowing for simultaneous excitation of different types and different color emitting QDs by one single monochromatic source. Furthermore, QDs were demonstrated to have narrow, symmetric, and size-tunable emission spectra, high extinction coefficients and quantum yields (Zhang and Clapp 2011), the latter reaching up to 50 % (Nam et al. 2011; Zhang and Zhong 2011; Wang et al. 2012), long fluorescence lifetimes and a high resistance to physical and chemical degradation. Correspondingly, the field of potential photo-optical applications is very broad. In clinical diagnostics QDs have been used for example as fluorescence markers for ex vivo detection and imaging of cancer cells (Juzenas et al. 2008; Nida et al. 2005), as a specific marker for healthy and diseased tissues (Rotomskis 2008), for labeling healthy and cancerous cells in vivo (Gao et al. 2004), treatment of cancer by photodynamic therapy (Bakalova et al. 2004), or sensing of the neurotransmitter dopamine (Zhao et al. 2013).

The application of colloidal semiconductor QDs in optoelectronic devices, such as light emitting diodes (LEDs) or photovoltaic cells, makes use of their largely tunable band gaps and durability (Yuan et al. 2012). QD-based light-emitting diodes benefit from the possibility to control color transitions which allows for generating colors that appear much purer than in the case of conventional semiconductor LEDs (Sun et al. 2007).

Besides size, composition is another key parameter that largely impacts not only the opto-electronic properties of QDs (quantum yield, PL intensity, emission range and maximum), but also their acceptance by potential users. While QDs of the first generation composed of Cd, Se, Pb or Te (Lee et al. 2000; Althues et al. 2006) were facing serious health concerns because of their toxicity along with the general unease towards inhalable nanoparticles, their successors composed of heavy metal-free group Ia, III and VI elements, such as CuInS_2_ (Chen et al. 2011) and CuInSe_2_ (Bailey and Flood 1998; Schock and Noufi 2000; Contreras et al. 1999; Nanu et al. 2004) are considerably less toxic and have a very large absorption coefficient. Alloying of CuInS_2_ with Zn allows for an even better tuning of the broad emission wavelength of this type of QDs from visible to near IR (Yuan et al. 2012). Coating of core quantum dots with a protective layer is nowadays an established technique that prevents the sensitive surface of QDs from agglomeration which would negatively affect their photoluminescence (PL) characteristics (Dabbousi et al. 1997; Weaver et al. 2009). For CuInS QDs the QY increased for example from ca. 5.4 to ca. 50 % after coating the core particles with a ZnS shell (Nam et al. 2011).

Covalent immobilization of QDs on the surface of a suitable matrix is a prerequisite to many applications, and is a means of reducing the health risk related to respirable particulate matter, too. By grafting QDs onto the large inner surface of lightweight aerogels, novel functional materials advantageously employing the large interconnected porosity and void surface area can be created. Next to sensor, opto-electronic or photovoltaic applications, QD containing aerogels could be used as true volumetric 3D displays as it has been recently successfully demonstrated for CdSe/ZnS-silica hybrid aerogels (Marinov et al. 2010). The true static 3D image—generated inside a suitable cube by simultaneous excitation of different types of QDs by a single focused beam from infrared lasers with different wavelengths and intensities—can be viewed from all or most of its sides at full preservation of its ultimate physiological depth cues. Direct volumetric displays are based on an “addressable volume of space created out of active elements (QDs) that are transparent in the *off* state but are either opaque or luminous in the *on* state” (Marinov et al. 2010).

Grafting of QDs onto the large surface of aerogels is possible if the respective QDs are furnished with suitable functional groups that can form covalent linkages with the solid aerogel network structure. However, synthesis of QDs through thermolysis in high-boiling solvents is commonly accomplished by simultaneous introduction of non-polar, hydrophobic ligands to support surface deactivation and to prevent QDs from agglomeration which would negatively impact their photoluminescence properties. Hence covalent immobilisation of QDs on the surface of solids requires the introduction of moieties that carry respective anchor groups. This can be achieved either by inclusion of hydrophobic QDs into amphiphilic micelles leading to an interdigitated bilayer (Dubertret et al. 2002) or by ligand replacement (Chan and Nie 1998).

Different from the bilayer approach where the supramolecular assembly is mainly maintained by local hydrophobic interactions, ligand replacement—for example by mercapto-functional groups—allows for establishing much stronger linkages between the QDs and the bridging ligands used for covalent grafting (Dubois et al. 2007). Replacement of ligands has been reported for different types of QDs, such as those composed of CdSe/ZnS (Dubois et al. 2007; Park et al. 2010; Yang and Zhou 2011) or CuInS/ZnS (Kim et al. 2011). For CuInS/ZnS (core/shell) QDs that were obtained by thermolysis of respective salts and 1-mercaptododecane in octadecene at 210 °C, a 60 % replacement of the original mercaptododecyl by α-mercapto-ω-hydroxyundecyl ligands was obtained when the exchange was performed immediately after completion of the growth of the core particles (Kim et al. 2011). The photoluminescence characteristics were fully preserved throughout ligand exchange as demonstrated for multilayer CdSe/CdS/CdZnS/ZnS QDs of which the trioctylphosphine oxide ligands were replaced by dithiocarbamate moieties (Dubois et al. 2007).

Covalent immobilisation of QDs equipped with ligands that contain terminal anchor groups on the surface of highly porous materials, such as aerogels, can be accomplished in two ways by synthesizing QD aerogels from sols of quantum dots, or by embedding prefabricated QDs in the supporting aerogel matrix of another material (Marinov et al. 2010).

To the best of our knowledge, the current paper describes for the first time the direct replacement of a considerable percentage of long-chain mercaptoalkyl groups introduced during thermolysis to prevent QDs from agglomeration by α-mercapto-ω-trialkoxysilyl ligands at room temperature, the furnishing of alloyed (ZnS)_x_(CuInS_2_)_1−x_/ZnS (core/shell) QDs with ligands having terminal functionalities that can be used as anchor groups for covalent immobilization on cellulose, and eventually the preparation of highly open-porous, lightweight, fluorescing cellulose aerogels with covalently immobilized QDs with high quantum yields of up to 30 % and emission colors within a wide range of the visible light.

## Materials and methods

### Materials

Eucalyptus pre-hydrolysis kraft pulp (hwPHK; TCF bleached; MW 80.3 kg mol^−1^, CCOA 4.7 μmol g^−1^ C=O, FDAM 8.8 μmol g^−1^ COOH; (Liebner et al. 2009). Octadecene, 1-mercaptododecane, toluene, CuI, In(OAc)_3_, Zn(OAc)_2_, 1-hexyl-3-methyl-1*H*-imidazolium chloride (HMImCl) and 3-(mercaptopropyl) trimethoxysilane (MPtMS) were purchased from Sigma-Aldrich (Sigma-Aldrich HandelsGmbH, Vienna, Austria). Pressurized CO_2_ was purchased from Linde Gas, Austria.

Synthesis of alloyed (ZnS)_x_(CuInS_2_)_1−x_ core/ZnS shell QDs of variable shell thickness and 1-mercaptododecane ligands grafted onto the particle surface was accomplished as described elsewhere (Nam et al. 2011). In brief, alloyed (ZnS)_x_(CuInS_2_)_1−x_ core QDs of different composition (Table 1) were obtained by reductive co-thermolysis of CuI, In(OAc)_3_, Zn(OAc)_2_ in the presence of a large excess of 1-mercaptododecane at 230 °C using 1-octadecene as high-boiling, non-coordinative solvent. Copper (I) iodide instead of copper acetate was used as the former has been demonstrated to afford a narrower QD size distribution (Li et al. 2009).

**Table 1:** Types of quantum dots used in the current study

QD (λ_em,max_)	Core composition	Core growth time at 230 °C (min)
QD_537_	Zn_0.7_In_1.0_Cu_1.0_S	60
QD_565_	Zn_0.7_In_2.0_Cu_1.0_S	60
QD_594_	Zn_0.5_In_2.0_Cu_1.0_S	60
QD_622_	Zn_0.2_In_2.0_Cu_1.0_S	60
QD_660_	Zn_0.5_In_4.0_Cu_1.0_S	120

Lower case numbers indicate the QDs’ emission wavelength (in toluene) and ratio of core-forming elements. All QDs were coated with ZnS by adding a total of 0.8 mL Zn(OAc)_2_ at 240 °C in two equal portions, allowing a reaction time of 30 min after each step

Following the preparation of alloyed (ZnS)_x_(CuInS_2_)_1−x_ core particles, shell formation was accomplished by raising the temperature to 240 °C, adding 0.8 mL of Zn(OAc)_2_ in two equal portions and allowing a reaction time of 30 min after each step. Similar to the core particles, shell formation was accomplished by co-thermolysis of Zn(OAc)_2_ and an 80fold excess of 1-mercaptododecane. A smaller excess of octadecylamine was also added, as alkyl amines have been shown to amplify the protective effect of QD coating and afford stable and reproducible luminescence quantum efficiencies of about 50 % at room temperature (Talapin et al. 2001).

Purification of QDs was accomplished by a sequence of colloidal dispersion/centrifugation/discarding the precipitate/QD precipitation from the supernatant using acetone/centrifugation that was repeated four times. In brief: QDs were dissolved in toluene and insoluble components were separated by centrifugation at 6,000 rpm (12 min) and discarded. Acetone was added to the toluene phase to re-precipitate QDs, and the QDs were separated by centrifugation. For compatibilization of the QDs with ionic liquids and covalent binding onto the cellulose surface, 1-mercapto-*n*-dodecyl ligands were replaced by MPtMS via phase transfer. In brief, a solution of 0.05 mL of ΜPtMS in 5.0 mL of HMImCl was added to 5.0 mL of a solution that contained the (ZnS)_x_(CuInS_2_)_1−x_/ZnS (alloyed core/shell) nanoparticles solubilized in toluene by the presence of lipophilic 1-mercapto-*n*-dodecyl ligands. The resulting two-phase system was vigorously stirred at ambient temperature for 30 min whereupon the QDs moved from the supernatant toluene into the lower ionic liquid phase which was then separated from the supernatant.

### Preparation of cellulose-(ZnS)_x_(CuInS_2_)_1−x_/ZnS (core/shell) QD composite aerogels

Eucalyptus prehydrolysis kraft pulp (hwPHK) was dissolved in HMImCl at 100 °C to afford solutions that contained 1–3 wt% of cellulose. Based on microscopic evaluation (Novex B series binocular microscope BBS Led for bright field contrast) full dissolution was assumed to be achieved within 2 h for all variants. The solutions were cooled down to 60 °C before different aliquots of the suspension of the 3-(trimethoxysilyl)-propyl-functionalized (ZnS)_x_(CuInS_2_)_1−x_/ZnS (core/shell) QDs in HMImCl were added dropwise under argon protection and vigorous stirring. Then, the solutions containing 0.01–0.3 wt% of QDs were transferred into PTFE molds. Disk-like alcogels (Ø 30 mm, height 3 mm) were obtained by coagulation of cellulose with either absolute or aqueous (50 v%) ethanol and replacing the coagulation media by fresh solvent every 4 h for three times at least. If aqueous ethanol was used for coagulation, the samples were thoroughly equilibrated in absolute ethanol prior to scCO_2_ drying (three times, 4 h each).

#### Supercritical CO_2_ drying

The composite alcogels were placed onto stainless steel filter panels inside the autoclave (SFP-200, Separex, Champigneulles, France). The system was then pressurized through the bottom valve with liquid, pre-heated CO_2_ using a HPLC pump until the operation pressure of 10 MPa was reached. The top valve was opened and the bottom valve was subsequently switched to the separator, where ethanol and CO_2_ were separated by an isothermal flash. Drying was accomplished at 40 °C using a drying time of 5 h. Finally the top valve was closed and the autoclave was depressurized over the separator.

### Analytical methods

*ATR*-*FTIR* spectra (500–4,000 cm^−1^) of the pure and composite aerogel discs were recorded with a L128-0099 PerkinElmer Spectrometer (Waltham, MA, USA). *Fluorescence* experiments were conducted using a PerkinElmer LS55.

*Transition electron microscopic (TEM)* pictures of cellulose-(ZnS)_x_(CuInS_2_)_1−x_/ZnS (core/shell) QD composite aerogels were obtained with a JEM-2010 FEF (UHR; JEOL, Tokyo, Japan). *Scanning electron microscopy* *(SEM)*: Hitachi X-650. Gold sputtering (5 nm) was performed at a voltage of 2.5 kV under argon protection. *Energy*-*dispersive X*-*ray* *(EDX):* Horiba EX-250 coupled with SEM. *X*-*ray diffractometry* of cellulose and cellulose-(ZnS)_x_(CuInS_2_)_1−x_/ZnS composite aerogels was performed in reflection mode (Rigaku RINT 2000, Japan) using monochromatic Cu K_α_ radiation (*λ* = 0.15406 nm).

*Nitrogen sorption experiments* at 77 K were conducted on a Micrometrics ASAP 2020 analyzer. Specific surface areas were calculated from the BET equation, the average pore diameter being evaluated by the BJH equation on the desorption branch of the isotherm. All samples were kept under vacuum overnight prior to the measurements. *Thermogravimetric* *analysis* *(TGA)*: NETZSCH TG209 F1. A constant heating rate of 20 °C min^−1^ was used throughout the entire temperature range studied (25–800 °C). The mechanical response to compression stress was investigated with a Zwick/Roell Materials Testing Machine Z020. A 50 N load cell was used to measure the force required to achieve a deformation rate of 2.4 mm min^‐1^.

^*1*^ *H,* ^*13*^ *C, and* ^*29*^ *Si solution state NMR spectroscopy* was performed on a Bruker Avance II 400 spectrometer with a 5 mm broadband probe head equipped with z-gradient in DMSO-d6 (^1^H frequency 400.13 MHz, ^13^C: 100.61 MHz, and ^29^Si: 79.49 MHz) at room temperature with standard Bruker pulse programs. Chemical shifts are given in values of ppm, referenced either to residual solvent signals (2.49 for ^1^H, 39.6 for ^13^C) or TMS (0.00 for ^29^Si), respectively. ^1^H NMR data were collected with 32 k data points and apodized with a Gaussian window function. ^29^Si NMR data were acquired with the DEPT sequence and WALTZ 16 ^1^H decoupling using 64 k data points. Signal-to-noise enhancement was achieved by multiplication of the FID with an exponential window function (lb = 3 Hz). Edited ^1^H,^13^C HSQC spectra were recorded 1k × 256 data points, using adiabatic pulses for inversion and GARP decoupling of the carbons. ^1^H,^29^Si HMBC spectra were acquired with 1k × 128 data points and the ^29^Si-^1^H long-range coupling constant set to 8 Hz. Both 2D experiments were acquired with 16 transients, sweep widths and offsets were individually adjusted. The resulting FIDs were zero-filled to a 2 × 1k data matrix and apodized with a shifted cosine function in both dimensions prior to Fourier transformation.

## Results and discussion

Synthesis of (ZnS)_x_(CuInS_2_)_1−x_/ZnS (core/shell) quantum dots was accomplished by co-thermolysis (230 °C) of CuI, In(OAc)_3_, Zn(OAc)_2_ and 1-mercaptododecane in octadec-1-ene as the high-boiling solvent. The presence of an excess of 1-aminooctadecane and 1-mercaptododecane loosely grafted onto the surface of the QDs by electrostatic interaction promote surface deactivation and impart good dispersibility in non-polar liquids, such as toluene.

In a second step, the core QDs were thermolytically (240 °C) coated with a ZnS shell of controlled thickness by adding Zn(OAc)_2_ and octadecylamine to the solution that contained the core QDs and a large excess of 1-mercaptododecane in octadec-1-ene. Shell formation complements the deactivation effect of lipophilic ligands and prevent the QDs from excessive agglomeration which would inevitable negatively impact their photoluminescence properties.

The preparation of cellulose-quantum dot hybrid aerogels can be accomplished by two approaches: (a) Loading of quantum dots onto alcogels prepared beforehand and (b) dispersing QDs in a cellulose solution and subsequent coagulation of cellulose from that solution by addition of a cellulose anti-solvent. While approach (a) has been subject of a simultaneous study, this study focused on approach (b). Molecularly dispersed solutions of cellulose are crucial factors in this approach. However, it is difficult to find a direct cellulose solvent that simultaneously features a sufficiently good compatibility with lipophilic additives, such as the suspension of 1-mercaptododecyl-capped (ZnS)_x_(CuInS_2_)_1−x_/ZnS (core/shell) QDs in toluene. Ionic liquids, such as BMImCl or HMImCl, have a high cellulose dissolving performance, but they are immiscible with toluene. Thus, the 1-mercaptododecyl-endcapped (ZnS)_x_(CuInS_2_)_1−x_/ZnS (core/shell) QDs did not mix and remained in the upper toluene layer. Exchange of 1-mercaptododecane ligands by 1-mercapto-3-(trimethoxysilyl)-propyl ligands was shown to facilitate the transfer of QDs from the supernatant toluene into the lower ionic liquid phase. The occurrence of a ligand exchange followed by QD phase transfer is evident from the color transfer between the two phases and was confirmed by GC/MS analysis of the toluene phase that revealed an increasing amount of 1-mercaptododecane.

Replacement of 1-mercaptododecyl- by 1-mercapto-3-(trimethoxysilyl)-propyl ligands allows not only for a homogenous dispersion of the QDs in the cellulose/HMImCl solution, it also enables the QDs to get covalently immobilized on the large inner surface of the cellulosic aerogels. This is considered to be advantageous for two reasons: it reduces the potential health risk immanent to nanoparticles and renders the quantum dots more resistant towards extraction during scCO_2_ drying.

**Fig. 1:**
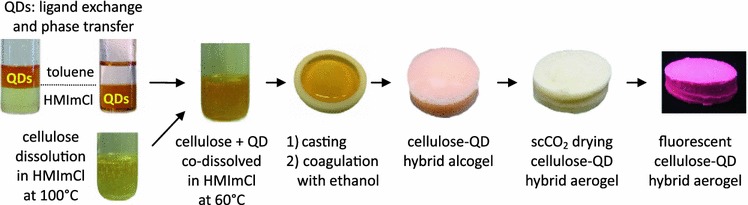
Schematic presentation of the approach to photoluminiscent cellulosic aerogels containing covalently grafted (ZnS)_x_(CuInS_2_)_1−x_/ZnS (core/shell) quantum dots

Figure 1 summarizes the steps required to obtain cellulose aerogels that contain covalently immobilized, capped (ZnS)_x_(CuInS_2_)_1−x_/ZnS (core/shell) quantum dots: (a) joint dissolution of 1, 2, or 3 wt% of cellulose (ca. 60 min) and dispersing 0 or 0.3 wt% of QDs in HMImCl at 60 °C, (b) casting, (c) coagulation of cellulose by adding ethanol, and d) converting the alcogels into aerogels using supercritical carbon dioxide (40 °C, 10 MPa).

**Fig. 2:**
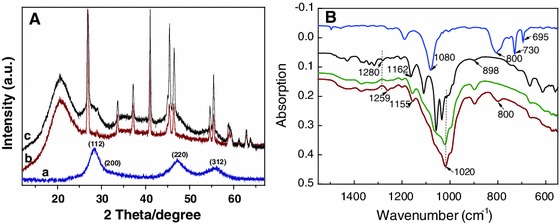
**a** XRD spectra of pure QDs (*a*), a QD-free cellulose aerogel (*b*), and a cellulose-QD hybrid aerogel (*c*). **b** FT-IR of QDs capped with MPTmS (*a*), cellulose aerogel (*b*) and two cellulose-QD hybrid aerogels containing 0.06 wt% (*c*) and 0.3 wt% (*d*) of surface-grafted (ZnS)_x_(CuInS_2_)_1−x_/ZnS (core/shell) quantum *dots*

Covalent linkage of 1-mercapto-3-(trialkoxysilyl)-propyl-capped QDs to cellulose is assumed to occur mainly upon dispersing the QDs in the cellulose solution in HMImCl at 60 °C (60 min). Surface silanisation of cellulose nanocrystals with 3-aminopropyltrimethoxysilane has been shown to occur at room temperature already (Yang and Pan 2010), and reaction with the cellulose molecules in solution can reasonably be assumed to be even faster. Covalent binding of 3-mercaptopropyl-trimethoxysilane onto the cellulose surface is evident from the permanent color transfer from the QD-containing ionic liquid to the regenerated cellulose, the absence of quantum dots in the separator unit after scCO_2_ drying of the cellulose aerogel/QD composites, and the EDX spectra of the products. Alcogels from bacterial cellulose loaded with 1-mercapto-3-(trialkoxysilyl)-propyl-capped (ZnS)_x_(CuInS_2_)_1−x_ alloyed core/ZnS shell quantum dots and subsequently scCO_2_-dried clearly showed the presence of silicon (data not shown). XRD spectra (Fig. 2a) also confirm the presence of QDs in the aerogels as the diffraction peaks at 2θ = 28.2°, 47.5°, and 56.0° typical for the (112), (220), and (312) crystallographic planes of the cubic (ZnS)_x_(CuInS_2_)_1−x_ lattice.

A comparison of the FT-IR spectra (Fig. 2) of purified MPTmS-capped QDs (a), pure cellulose aerogel (b), and cellulose/QD composite aerogels (c,d) provides further evidence of the covalent immobilization of the QDs onto cellulose. While MPTmS shows two typical absorption peaks at 1,080 cm^−1^ (*Si*–*O*–C) and 800 cm^−1^ (Si–*O*–*C*) caused by the stretching vibrations of the silyl-ether bonds (Primeau et al. 1997), one additional band due to stretching vibrations in the newly formed cellulose-silicon ethers was observed at 1,020 cm^−1^ (*Si*–*O*–C), its intensity increasing with the amount of QDs added. Grafting of QDs onto the cellulose is particularly evident from the shift and line broadening of the absorption peaks at 1,280 and 1,162 cm^−1^ (pure cellulose, C–O-C stretching vibrations) towards 1,259 and 1,155 cm^−1^ occurring with the addition of QDs. It might be speculated that this is due to (p → d)π interactions between silicon covalently attached to cellulose via ether bonds preferably (see below) in C6 position of the anhydroglucose units and the ring oxygen atoms of the cellulose backbone.

**Fig. 3:**
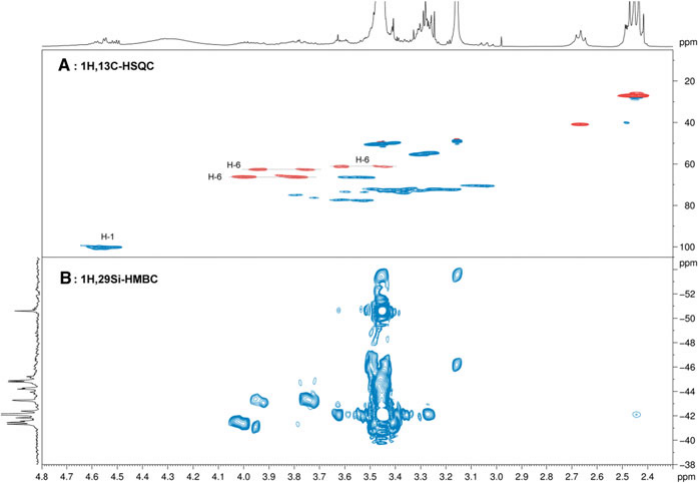
Gradient-selected ^1^H,^13^C HSQC and long-range ^1^H-^29^Si HMBC NMR spectra of the reaction mixture obtained from methyl-D-glucopyranoside and 3-mercptopropyl-trimethoxy-silane at 60 °C and 60 min reaction time. **a** ^1^H,^13^C-HSQC, different phases for CH_2_ (*red*) and CH and CH_3_ (*blue*). **b** ^1^H,^29^Si-HMBC for detection of long-range couplings

Covalent binding of the 1-mercapto-3-(trialkoxysilyl)-propyl-capped (ZnS)_x_(CuInS_2_)_1−x_/ZnS (core/shell) quantum dots onto cellulose was indirectly confirmed by liquid-state NMR using methyl-D-glucopyranoside as a cellulose model compound and 3-mercaptopropyl-trimethoxysilane instead of the MPTmS-capped QDs. ^29^Si NMR spectra of the reaction mixture treated at 60 °C (temperature of cellulose dissolution in HMImCl) for 60 min contained a couple of resonance signals in the range of −40 to −54 ppm in addition to that one caused by MPTmS itself (δ = −42.1 ppm, reference: TMS). 2D NMR experiments were performed to prove whether the above newly formed organosilicon compounds are silyl ethers of methyl-D-glucopyranoside or just self-condensation products. Gradient-selected ^1^H-^13^C HSQC experiments proved the etherification by the typical down-field shifts of the resonances in both the ^1^H and the ^13^C domain (Fig. 3a). The presence of long-range ^1^H-^29^Si HSQC cross-peaks (set *J*_H,Si_ = 10 Hz, Fig. 3b) in exactly that region and the absence of the educt methyl-D-glucopyranoside in the reaction mixture after 60 min reaction time at 60 °C are clear evidence of the formation of respective silyl ethers of the methyl glucoside. The spectra furthermore indicate that the primary hydroxy group (OH-6) is a main binding site for the silyl coreactant.

**Fig. 4:**
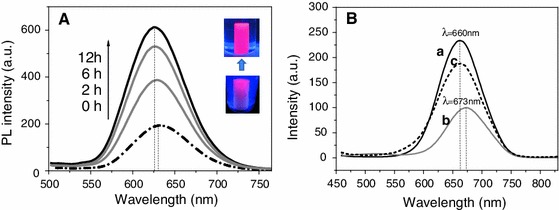
**A** Photoluminiscence spectra (λ_ex_ = 400 nm) taken during coagulation of cellulose from a respective solution in HMImCl (2 wt% hwPHK) that additionally contained 0.12 wt% of QD_622_. Absolute ethanol was used as cellulose anti-solvent, and was replaced three times every 4 h. Insert: Illustration of the increasing PL intensity of alcogels during coagulation. **B** PL spectra of a suspension of 0.5 mg mL^−1^ of 1-mercaptododecyl-capped QD_660_ in toluene (*a*), HMImCl (*b*) and ethanol (*c*)

Coagulation of cellulose which has been accomplished by addition of the cellulose anti-solvent ethanol can be monitored by fluorescence spectroscopy as the increasing dilution of the ionic liquid HMImCl by ethanol causes a strong increase of the PL intensity with its maximum value being reached when all ionic liquid is replaced by ethanol. Compared to the initial PL intensity of HMImCl that contained 2 wt% of dissolved cellulose and 0.12 wt% of dispersed QD_622_ for example (*t* = 0, *T* = 25 °C), the respective value was approximately twice as high already after 2 h of cellulose coagulation (Fig. 4a). Repeated replacement of diluted IL by ethanol during an additional time period of 10 h further increased the PL intensity arriving at about 300 % compared to the initial value. Based on the observed sensitivity of the surface grafted QDs towards changes of the surrounding fluid, fluorescence spectroscopy can be used to determine the endpoint of solvent exchange—and hence cellulose coagulation—which is reached if addition of anti-solvent does no longer increase the PL intensity.

The maximum emission wavelength of QDs does not only depend on the nature and stoichiometric ratio of their constituents, size, core/shell ratio or type and concentration of ligands, it also depends on interactions of the QDs with their chemical environment. While a suspension of 1-mercaptododecyl-capped (ZnS)_x_(CuInS_2_)_1−x_/ZnS (core/shell) QDs of a Zn_0.5_Cu_1.0_In_4.0_ core constitution (120 min core growth, 230 °C) in toluene (0.5 mg mL^−1^) has its emission maximum at 660 nm, λ_em_ shifts to 673 nm after ligand exchange and phase transfer of the QDs from toluene to HMImCl. As soon as the majority of the ionic liquid is replaced by ethanol, λ_em,max_ shifts back to 660 nm (Fig. 4b).

**Fig. 5:**
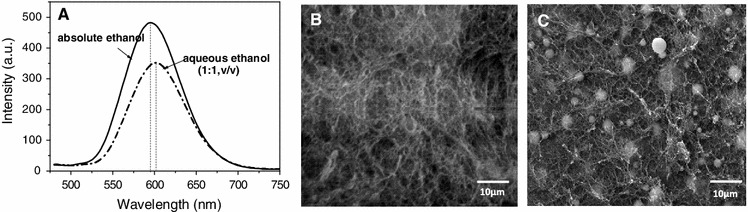
PL spectra (**a**) and SEM pictures (**b**, **c**) of cellulose hybrid aerogels containing (ZnS)_x_(CuInS_2_)_1−x_/ZnS (core/shell) QDs differing from each other by the anti-solvent used for cellulose coagulation from solution state (HMImCl, 2 wt% cellulose, 0.12 wt% QD_594_; B: absolute ethanol, C: 50 v % aqueous ethanol). After cellulose coagulation the respective gels were stored in absolute ethanol for another 12 h prior to scCO_2_ drying

The presence of water during the preparation of cellulose-QD hybrid aerogels should be avoided as it does not only impede cellulose dissolution in HMImCl, but also promotes self-condensation of QDs, which is known to reduce the PL intensity significantly (Artemyev et al. 2000; Yu et al. 2008). Similarly, the presence of water during cellulose coagulation from HMImCl has been demonstrated to have a negative effect on both aerogel homogeneity and PL properties. Large aggregates randomly distributed across the cellulose-QD hybrid network were formed when water was present during coagulation (Fig. 5c). This is assumed to be due to the formation of silanol groups from alkoxysilyl moieties not involved in QD grafting onto cellulose during the one-hour residence time of QDs in the cellulose solution (60 °C) prior to coagulation. Cellulose-QD aggregate formation and deposition of particles was found to cause a slightly bathochromic PL shift (λ_em,max_ = 592 → 598 nm) of the final aerogels. Furthermore, PL quenching (see above) dropped the fluorescence intensity by about 25 % compared to aerogels that were obtained by regenerating cellulose with absolute ethanol (Fig. 5a).

In contrast, particle formation was almost close to zero when absolute ethanol was used for cellulose coagulation, and the PL intensity was almost as high as for the respective alcogels. Therefore, absolute ethanol is recommended as suitable anti-solvent for the preparation of this particular type of cellulose-QD hybrid aerogels.

**Fig. 6:**
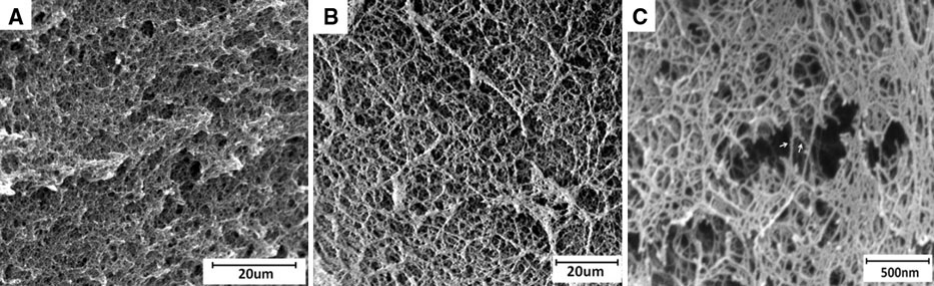
Scanning electron micrographs: Inner sections of a pure cellulose aerogel (**a**) and of a cellulose hybrid aerogel that contained covalently linked QD_565_ (**b**, **c**). Both materials were obtained from 2 wt% cellulose containing solutions in HMImCl, the amount of QD added was 0.12 wt%

Scanning electron micrographs (SEM) of cross-sections confirm that the morphology of cellulose aerogels in terms of solid network structure and macropore characteristics which were virtually not affected by grafting of 1-mercapto-3-(trialkoxysilyl)-propyl-capped QDs onto cellulose (Fig. 6a–c). The presence of (ZnS)_x_(CuInS_2_)_1−x_/ZnS (core/shell) QDs on the surface of the cellulose network structure is visible on SEM pictures of higher magnification (Fig. 6c).

**Fig. 7:**
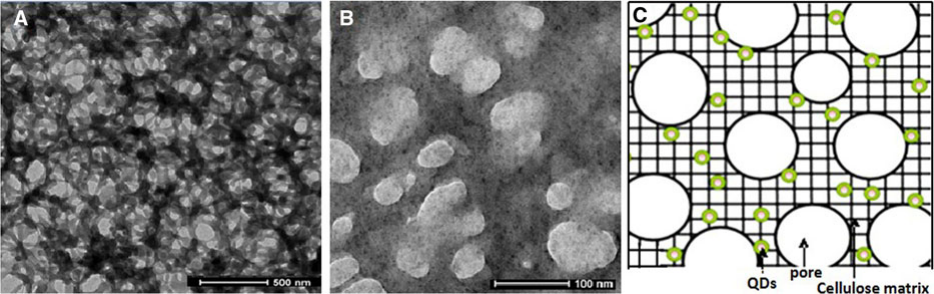
TEM images of cellulose-(ZnS)_x_(CuInS_2_)_1−x_/ZnS (core/shell) QD hybrid aerogels confirming the presence of quantum dots having an average diameter of about 15 nm (**a**, **b**). Schematic representation of QD distribution across the solid cellulose network structure (**c**)

A largely uniform distribution of the QDs within the cellulosic matrix has been also confirmed by transmission electron microscopy (TEM) as shown in Fig. 7. It is evident that a considerable fraction of the QDs has been rather closely embedded into the cellulose network structure during coagulation while the remaining part of the QDs is located on the surface of the fibrous aggregates and are hence part of the void surface (cf. scheme C). This is similar to materials obtained by Luong et al. (2008) who studied the formation of silver nanoparticles inside a nanofibrous cellulose acetate aerogel by reduction of silver nitrate with NaBH_4_.

**Fig. 8:**
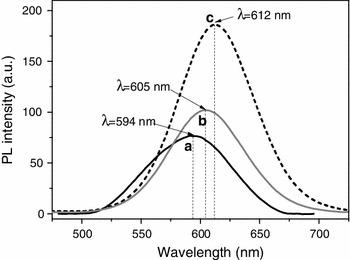
Photoluminiscence spectra (λ_ex_ = 470 nm) of a suspension of 1-mercapto-dodecyl-capped QD_594_ (0.125 mg mL^−1^) in toluene (**a**) and of cellulose hybrid alcogels that were obtained from a 2 wt% cellulose solutions in HMImCl the latter containing different amounts of 1-mercapto-3-(trimethoxysilyl)-propyl-capped QD_594_ (b: 0.12 wt%; c: 0.30 wt%)

The PL intensity of both cellulose-(ZnS)_x_(CuInS_2_)_1−x_/ZnS (core/shell) QD hybrid alcogels and aerogels can be controlled by the amount of QDs covalently grafted onto cellulose (Fig. 8). Increasing the amount of QD_594_ from 0.12 to 0.3 wt% dispersed in a solution of 2 wt% of cellulose in HMImCl for example resulted in 80 % PL intensity gain for the respective alcogels.

**Fig. 9:**
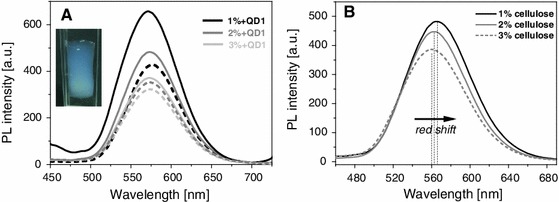
**A** Photoluminiscence spectra (λ_ex_ = 380 nm) of the top (*straight lines*) and bottom surfaces (*dotted lines*) of cylindrical cellulose-QD_565_ hybrid *alcogels* obtained from HMImCl solutions of different cellulose but fixed content (0.12 wt% in HMImCl) of quantum dots. **B** Impact of scCO_2_ drying and cellulose density on the photoluminescence of cellulose-hybrid *aerogels* obtained from the respective cellulose-QD_565_ hybrid alcogels (cf. 9A)

Compared to suspensions of the above type of purified QD_594_ which have their emission maximum in toluene at 594 nm, a slight red shift of the maximum emission wavelength was observed for some of the respective cellulose-QD hybrid alcogels which increased with the amount of QDs added (cf. Fig. 8, alcogel with QD_594_). A similarly weak red shift response towards changes of the QD/cellulose ratio was also seen for some of the aerogels, even though the overall blue shift caused by scCO_2_ drying was much more pronounced for all gels (cf. discussion below). A red shift of ≤8 nm for example was observed for cellulose-QD_565_ hybrid aerogels when reducing the cellulose concentration in HMImCl from 3.0 to 1.0 wt% which corresponds to an increase of the QD/cellulose ratio (Fig. 9B).

The occurrence of this phenomenon in anhydrous ethanol, where self-condensation of alkoxysilyl groups can be excluded evidences that increasing QD/cellulose ratios favor the aggregation of QDs after addition to the cellulose solution in HMImCl. Van der Waals interactions between the lipophilic mercaptododecyl- and aminooctadecyl-substituents that remained on the surface of the QDs after ligand exchange are supposed to be the driving force for this process. The negligible loss of QDs during scCO_2_ drying furthermore suggests that most of the QDs involved in agglomeration are covalently linked to cellulose which hence additionally contributes to aggregate formation.

Aggregation of QDs is a well-known reason for PL quenching and a red shift of the optical transitions observed in absorption and excitation spectroscopy, either caused by delocalization and formation of collective electronic states (Artemyev et al. 2000) or by Förster resonance energy transfer (FRET) with its absolute value being a function of the QDs’ distance to each other. The occurrence of FRET due to QD aggregation has been described for example for ZnO QDs grown on the sidewalls of multiwalled carbon nanotubes (Dutta et al. 2010), epoxy/ZnO hybrid resins (Sun et al. 2008), PEO/CdS hybrid ultrafine fibers (Yu et al. 2008), and amphiphilic hybrid PS-block-PEO/TiO_2_/CdS thin films (Kannaiyan et al. 2010) which all contained QDs non-covalently linked to the respective polymer.

Grafting of homogeneously dispersed QDs onto the inner surface of polymeric network seems to be able to suppress the above red shift to a large extent as it is evident from the non-existent (*cf*. Fig. 9A) to very small red shift (cf. Figs. 8, 9B). This is in good agreement with other studies and has been shown for 1-thioglycerol-capped CdSe QDs embedded in a PMMA matrix (Artemyev et al. 2000), poly(maleic acid-alt-octadecene)-encapsulated CdSe/CdS/ZnS (core/shell/shell) quantum dots (Cho et al. 2013) or bacterial cellulose-(ZnS)_x_(CuInS_2_)_1−x_/ZnS (core/shell) QD—hybrid aerogels (data not shown).

The apparent density of the cellulose network structure is another factor that considerably affects the PL intensity of both alco- and aerogels. The decline of PL intensity that was observed when increasing the amount of cellulose dissolved in HMImCl and hence the density of the gels is assumed to be mainly caused by scattering losses and the above discussed quenching effects. For cellulose-QD_565_ hybrid aerogels, for example, a PL loss of about 20 % at λ_max_ (λ_ex_ = 380 nm) was observed after increasing the cellulose content in HMImCl from 1.0 wt% (ρ = 37.9 mg cm^−3^) to 3.0 wt% (ρ = 57.2 mg cm^−3^; Fig. 9B).

Interestingly, an anisotropic PL response was observed for all cylindrical alcogels. Front surfaces of the cylindrical bodies that were bottom down during shaping/coagulation of cellulose from respective solutions in HMImCl exhibited a higher PL intensity compared to the top faces all cylindrical samples. This might by surprising at a first glance as the cellulose density of the gels increases from top to bottom. According to the impact of cellulose density (cf. Fig. 9B), a reduced PL intensity would have to be expected for the lower parts of the samples. However, the contrary observation can be explained by two effects: (a) precipitation of cellulose aggregates which is pronounced in the initial state of cellulose coagulation that starts from the top of the samples as the latter were covered with the cellulose anti-solvent for coagulation; (b) replacement of HMImCl by the antisolvent ethanol leads to a downward-moving phase border with increased solute concentration in the HMImCl phase. Covalently bound to cellulose during the dispersing step (60 °C, 1 h), QDs are carried along towards the lower parts of the cast gel when cellulose precipitation sets in or when a cellulose gradient is induced by adding an anti-solvent. The sum of the above-described effects eventually causes higher absolute QD concentrations at the bottom of the samples even though the relative cellulose/QD ratio might remain unaffected. As the differences in transparency between the upper and lower parts of the respective alcogels are on the other hand very small, the higher QD content at the bottom is hence responsible for the higher PL intensity observed here (Fig. 9A).

The differences in PL intensity between top and bottom sections of the samples were shown to be a function of cellulose concentration in HMImCl. While a considerable reduction in PL intensity was observed for the top layers of those samples that were obtained from 1 wt% cellulose containing solutions in HMImCl, the differences were much smaller when increasing the solute concentration to 3 wt%. This is assumed to be due to the increasing viscosity of the cellulose solution that impedes precipitation of regenerated cellulose-QD aggregates.

A far reaching homogeneous distribution of the QDs across the aerogels under preservation of an acceptably high PL can be hence achieved by using a sufficiently high cellulose concentration (≥3 wt%) in HMImCl, an appropriate amount of QDs to compensate for the decreasing transparence of the materials or by preparation of samples (films, disks etc.) with thicknesses not exceeding 5 mm.

Supercritical CO_2_ drying as it is applied to convert alcogels into aerogels has been demonstrated to maintain the PL characteristics of the parent materials to a large extent. This was shown for alcogels obtained from 3 wt% cellulose dissolved in HMImCl, as the PL characteristics of these particular samples feature a far-reaching homogeneity across the sample profile. As in the case of the alcogels for which a slight red shift was observed when increasing the amount of QD at constant cellulose content, a similarly weak red shift of about 6 nm (λ_em,max_) occurred when the amount of cellulose dissolved in HMImCl dropped from 3 to 1 wt% (cf. discussion above). However, this faint impact of the QD/cellulose ratio is superimposed by a somewhat more pronounced overall blue shift caused by the scCO_2_ drying step. This effect that has been reported earlier (Amato et al. 1996), and it can be explained by quantum confinement resulting from shrinking and hence smaller nanostructures present in the scCO_2_-dried samples.

**Fig. 10:**
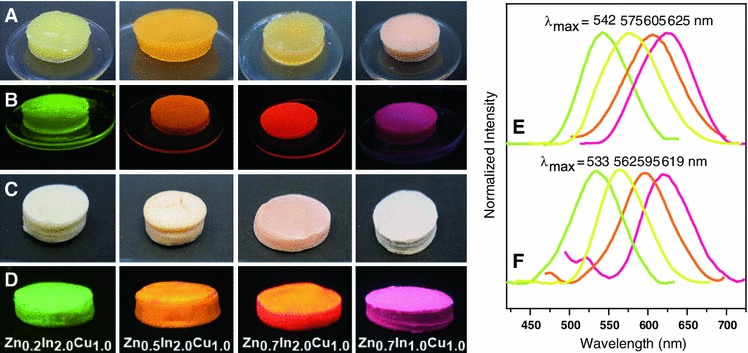
Pictures of cellulose–QD hybrid alcogels (**a**, **b**) and aerogels (**c**, **d**) taken under visible (**a**, **c**) and UV light (**b**, **d**). The materials were obtained from a solution of 2 wt% of cellulose in HMImCl containing 0.12 wt% of (ZnS)_x_(CuInS_2_)_1−x_/ZnS (core/shell) QDs of different composition (provided in d). Figure **e** and **f** show the respective fluorescence spectra of the alcogels prior to (**e**) and after (**f**) scCO_2_ drying

The photoluminescence of both cellulose hybrid alcogels and aerogels containing covalently immobilized (ZnS)_x_(CuInS_2_)_1−x_/ZnS (core/shell) quantum dots can be controlled over a wide range (460–710 nm) of the visible light by varying the stoichiometric composition of the QD core’s constituents, the core size (duration of particle growth) and the thickness of their ZnS shell. This is displayed in Fig. 10 which shows the pictures of alcogels and aerogels obtained from respective solutions of 2 wt% of cellulose in HMImCl that each contained 0.2 wt% of the different types of quantum dots. While the appearance of the respective alcogels (Fig. 10a, b) and aerogels (c, d) is rather non-spectacular when observed at daylight (a, c), their bright fluorescence colors covering the wide range from green to yellow to magenta fully develop under UV light (λ_ex_ = 367 nm; Fig. 10b, d).

Disk-like cellulose-QDs composite alcogels are largely transparent and feature a uniformly high fluorescence across the samples. This indicates a homogeneous distribution of the (ZnS)_x_(CuInS_2_)_1−x_/ZnS (core/shell) nanoparticles within the cellulosic network which is supported by SEM pictures as exemplarily shown in Fig. 6c. After scCO_2_-drying the obtained aerogels are off-white at daylight (Fig. 10c) tending to have a hue that corresponds to the color observed under UV light.

Grafting of QDs onto cellulose in solution state mediated by 3-(trimethoxysilyl)-propyl ligands protruding from the surface of the core/shell nanoparticles has been demonstrated to reduce the overall shrinkage that is commonly observed during solvent exchange and scCO_2_ drying. A better preservation of the hierarchical pore structure can be concluded when comparing the apparent densities of the most lightweight cellulose-QD hybrid aerogels with that of the QD-free counterparts. For all aerogels obtained from ≤2 wt% cellulose and 0.12 wt% QD_565_ (related to HMImCl), respectively, lower apparent densities compared to QD-free samples were obtained (Table 2; samples 1 % + QD1 and 2 % + QD1) even though the total amount of solutes in HMImCl was higher for the hybrid aerogels (QD + cellulose).

**Table 2:** Apparent densities, shrinkage during solvent exchange and scCO_2_ drying, porosity, pore surface areas, and mechanical characteristics of cellulose-(ZnS)_x_(CuInS_2_)_1−x_/ZnS (core/shell) QD hybrid aerogels

Sample	% in HMImCl		Aerogel density (g cm^−3^)	Remaining volume (%)*	Surface area (m^2^/g)	Calc. porosity (%)	Youngs modulus (MPa)	Yield strength σ_0.2_ (kPa)
	Cellulose	QD_565_						
1 % pure	1	0.00	0.0386	56	136	97.58 %	0.22	16.5
1 % + QD1	1	0.12	0.0379	67	358	n.d.	0.27	23.5
2 % pure	2	0.00	0.0432	60	350	97.25 %	0.27	20.3
2 % + QD1	2	0.12	0.0408	72	686	n.d.	0.34	27.4
2 % + QD2	2	0.30	0.0493	76	312	n.d.	0.69	55.2
3 % + QD1	3	0.12	0.0572	74	296	n.d.	0.79	65.3

* Remaining volume after coagulation and scCO_2_ drying

**Fig. 11:**
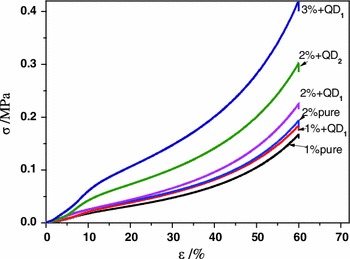
Mechanical response profiles of cellulose-QD aerogels (see Table 2) towards compressive stress

The enhanced preservation of the cellulose network structure is assumed to be due to cross-links between cellulose aggregates caused by the trifunctional terminal trialkoxysilyl groups of the QD capping ligands. According to the Gibson and Ashby model of cellular solids (Gibson and Ashby 1982), the mechanical properties of aerogels can be improved by reinforcing in particularly the edges of the cells, i.e. the joints of the cellulose fibrils that build-up the cellular frame. After reinforcement the aerogels showed an increased capability of absorbing energy by elastic deformation (cell wall buckling with the highest load impact in the center of the fibrils between two joints), which leads to higher stiffness (Youngs modulus) and strength (σ measured at 0.2 % off-set strain) as confirmed by the response profiles of the materials towards compressive stress (Table 2, Fig. 11). Similar as for bacterial cellulose or pulp-based aerogels, the obtained cellulose-QD hybrid aerogels do not suddenly collapse upon compression. The irreversible damaging of the cellular solid that happens once the flow limit is reached proceeds rather continuously with increasing compaction of the material. The Poisson ratio that describes the change of the cross-section area due to sample buckling during compression was close to zero. This is in good agreement with the mechanical response of aerogels that were previously obtained by coagulation of cellulose from 1-ethyl-3-methyl-1*H*-imidazolium acetate (Sescousse et al. 2011).

**Fig. 12:**
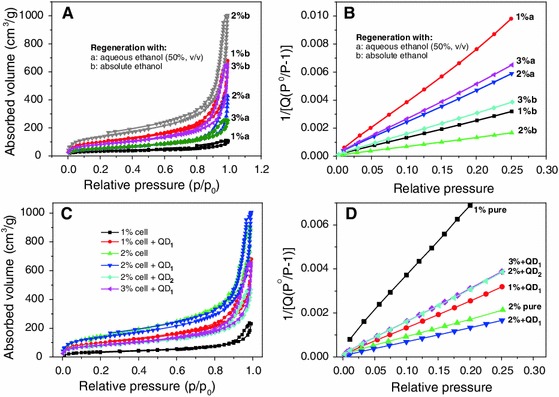
Results of nitrogen sorption experiments at 77 K. Impact of cellulose density, amount of QD_565_ grafted onto cellulose (**a,** **b**) and type of anti-solvent used for cellulose coagulation (**c**, **d**; 0.12 wt% of QD was used in all variants) on N_2_ sorption/desorption behavior (**a**, **c**) and the slope of the BET *curves* (**b**, **d**)

Nitrogen sorption experiments at 77 K evidenced that the open-porous network structure of the obtained cellulose-QD hybrid aerogels is dominated by a comparatively large fraction of mesopores. This has been concluded from the shape of the type IV isotherms (Fig. 12a, c) typical for mesoporous materials (Rouquerol et al. 1999). Similar as in the case of other cellulosic aerogels of comparable density, obtained for example by coagulation of cellulose from solution state (NMMO, e.g.), the shape of the hysteresis loops is in between the IUPAC classification types *a* and *b* referring to relatively narrow-sized mesopores (Rouquerol et al. 1999). This is largely in accordance with the SEM pictures which, however, also suggest the presence of a smaller fraction of macropores, most of them being smaller than one micron (cf. Fig. 6). The low intercepts of the BET curves (1 × 10^−3^–1 × 10^−4^; Fig. 12 b, d), however, evidence a rather small contribution of the macropores to the overall pore surface area which is in agreement with previous studies (Liebner et al. 2009, 2011). This is supported by the small N_2_ volumes adsorbed at *P*/*P*_0_ = 0.02 (approx. 2–100 cm^3^ g^−1^) which is considered to be the limit between mono- and multilayer adsorption (Fig. 12 a, c). The specific surface of the obtained cellulose-(ZnS)_x_(CuInS_2_)_1−x_/ZnS (core/shell) QD hybrid aerogels ranged from about 300–690 m^2^ g^−1^ which is in the same range as for their QD-free counterparts.

Coagulation of cellulose with aqueous ethanol was confirmed to afford materials of significantly smaller pore surface area compared to that obtained with pure ethanol (Fig. 12a, b). This is probably due to self-condensation of a considerable portion of the trialkoxysilyl-functionalized QDs leading to deposition of larger hydrophobic silica aggregates within the cellulosic network (cf. Fig. 5c) and even closure of some of the pores as observed previously (Litschauer et al. 2011).

Both scanning electron micrographs (Fig. 6) and N_2_ sorption experiments (Fig. 12) provide evidence that grafting of 1-mercapto-3-(trimethoxysilyl)-propyl-capped (ZnS)_x_(CuInS_2_)_1−x_/ZnS (core/shell) quantum dots onto cellulose in solution state does not negatively affect the morphology of the obtained hybrid aerogels. On the contrary, grafting of QDs were shown to have a reinforcing effect to the aerogels (see above) that provides them the capability to withstand contraction forces emerging during solvent exchange and scCO_2_ drying. This retains the accessibility of the void surface to a large extent which is evident from the high void (pore) surface areas of the 2 % + QD1 sample (686 m^2 ^g^−1^).

## Conclusions

(ZnS)_x_(CuInS_2_)_1−x_/ZnS (core/shell) quantum dots obtained according to the thermolytic approach have been successfully subjected to ligand exchange. Replacement of 1-mercaptododecyl- by 1-mercapto-3-(trimethoxysilyl)-propyl ligands allow for covalent binding of the respective QDs to cellulose. Co-dispersion of the 1-mercapto-3-(trimethoxysilyl)-propyl-capped (ZnS)_x_(CuInS_2_)_1−x_/ZnS (core/shell) quantum dots in solutions of cellulose in ionic liquids such as HMImCl at slightly elevated temperature (45–60 °C) and subsequent addition of a cellulose-anti-solvent affords largely transparent, fluorescent organogels. Their photoluminescence can be tuned within a wide range of the visible light by varying the composition of the core constituents, thickness of shell and the cellulose/QD ratio. Conversion of the organogels to aerogels by scCO_2_ drying preserves the PL properties to a large extent. The weak blue shift caused by scCO_2_ drying is superimposed by an even somewhat less pronounced bathochromic shift that occurs when increasing the amount of QDs at constant density of the cellulosic network. Grafting of (ZnS)_x_(CuInS_2_)_1−x_/ZnS (core/shell) quantum dots onto cellulose increases the mechanical stability of the hybrid aerogels compared to their QD-free counterparts which benefits high pore surface area, overall porosity, and dimensional stability of the samples during scCO_2_ drying. Cellulose organogels and aerogels containing covalently immobilized QDs of group Ia, III and VI elements are expected to have a large application potential and will be hence subject of further investigation.
